# Circular RNA circ_0076631 promotes coxsackievirus B3 infection through modulating viral translation by sponging miR-214-3p

**DOI:** 10.3389/fmicb.2022.975223

**Published:** 2022-09-06

**Authors:** Ying Qin, Lexun Lin, Shulong Yang, Zongmao Dai, Congcong Zhang, Jingjing Huang, Fengzhen Deng, Xinxin Yue, Long Ren, Yanru Fei, Wenran Zhao, Yan Wang, Zhaohua Zhong

**Affiliations:** ^1^Department of Microbiology, Harbin Medical University, Harbin, China; ^2^Department of Pediatric Surgery, The Second Affiliated Hospital of Harbin Medical University, Harbin, China; ^3^Translational Medicine Research and Cooperation Center of Northern China, Heilongjiang Academy of Medical Sciences, Harbin, China; ^4^Department of Cell Biology, Harbin Medical University, Harbin, China

**Keywords:** coxsackievirus B3, circRNA, miR-214-3p, 3D polymerase, viral replication

## Abstract

Coxsackievirus B (CVB), a member of *Enterovirus* genus of *Picornaviridae*, is the leading pathogen of viral myocarditis and dilated cardiomyopathy. The pathogenesis of CVB-induced myocarditis has not been completely elucidated, and no specific antiviral measurement is available presently. Circular RNAs (circRNAs) have been reported to be able to modulate viral replication and infection through bridging over non-coding RNAs (ncRNAs) and coding messenger RNAs (mRNAs). To date, the role of circRNAs in CVB infection is largely unknown. In this study, we found that hsa_circ_0076631 (circ_0076631) significantly promoted CVB type 3 (CVB3) replication. Further study showed that the underneath mechanism was circ_0076631 indirectly interacting with CVB3 through sponging miR-214-3p, which targeted the 3D-coding region of CVB3 genome to suppress viral translation. Knocking down circ-0076631 caused a suppression of CVB3 infection; thus, circ-0076631 may be a potential target for anti-CVB therapy.

## Introduction

Coxsackievirus group B (CVB) belongs to the human *Enterovirus* B species of *Picornaviridae* (Garmaroudi et al., 2015). Six serotypes have been identified in CVB (CVB1—CVB6). Among them, CVB1—CVB5 are the major pathogens of human viral myocarditis and dilated cardiomyopathy (DCM), and CVB3 is frequently used serotype for studying myocardial pathogenesis of CVB (Tian et al., 2018). CVB is also the pathogen of pancreatitis and aseptic encephalitis (Ye et al., 2013). Currently, there are no specific measurement to treat and prevent CVB infection. CVB genome is a positive single-strand RNA (+ssRNA). The genome is about 7.4 kb in length, including three functional regions: 5′ untranslated region (5′-UTR), a long open reading frame (ORF), and 3′-UTR (Souii et al., 2013; Honkimaa et al., 2020). The ORF is translated into a large polyprotein, which is cleaved into four structural and seven non-structural proteins by the viral proteinases 2A and 3C (Ben M’hadheb et al., 2015; Huang et al., 2017). The non-structural protein 3D plays as RNA-dependent RNA polymerase (RdRp) and is vital for CVB replication (Turkki et al., 2020).

The interaction between viral components and host cellular factor plays a key role in the pathogenesis of CVB (Kim et al., 2022). MiRNAs have been implicated in many viral infections via their mRNAs binding action (Trobaugh et al., 2017). Our previous studies showed that a variety of miRNAs can affect CVB pathogenesis. For example, miR-10a* can promote CVB replication (Tong et al., 2013), while miR-146a modulates the inflammatory response to CVB infection (Fei et al., 2020). Pyroptosis is a cell death process that is induced by proinflammatory signals. Our previous study demonstrated that pyroptosis is involved in the pathogenesis of CVB3 infections (Wang Y. et al., 2018).

Circular RNAs (circRNAs) are highly conserved non-coding RNAs in mammals with a length varied from fewer 100 to thousands of nucleotides (nt) (Pamudurti et al., 2017). CircRNA does not own 5′-cap structure and 3′-poly(A) structures and usually forms a closed circle (Beermann et al., 2016). CircRNA can bind to and “sponge” miRNA, and relieve the suppression of the miRNA on its target mRNAs and thereafter the gene expression (Beermann et al., 2016). Studies have shown that circRNAs are involved a variety of diseases, including cancer, inflammation, fibrosis, and viral infection (Memczak et al., 2013; Zhang H. D. et al., 2018; Zhang Z. et al., 2018; Yang et al., 2019; Xie et al., 2021). CircRNAs have been shown to regulate viral replication, viral pathogenesis, and antiviral immune response and thus may be candidate biomarkers and antiviral target for viral infection (Chen et al., 2017; Li et al., 2017; Sekiba et al., 2018; Tagawa et al., 2018; Wang S. et al., 2018; Yu et al., 2019; Zhao et al., 2019; Lu et al., 2020; Zhang and Wang, 2020). To date, the involvement of circRNAs in CVB replication remains unknown. Only hsa_circ_0000367 (circSIAE) has been reported to inhibit CVB3 replication by targeting cellular TAOK2 (thousand and one amino-acid kinase 2) through sponge adsorption of miR-331-3p (Yang et al., 2022).

Circ_0076631 has been reported to regulate the pyroptosis process of diabetic cardiomyopathy (Yang et al., 2019). In the present study, we found that circ_0076631 could promote the biosynthesis and replication of CVB3 by sponging miR-214-3p, suggesting that circ_0076631 may be a potential therapeutic target against CVB3 infection.

## Results

### Circ_0076631 abundance increases siginificantly in the CVB3-infected cells

**FIGURE 1:**
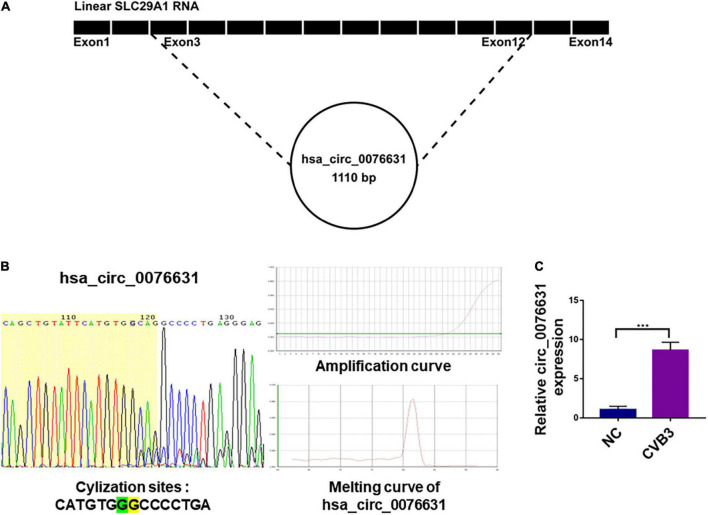
The source of circ_0076631 and association with CVB3 infection. **(A)** The genomic location of circ_0076631 in the transcript of SLC29A1 gene. **(B)** Ring formation by circ_0076631 was performed on the basis of the amplification curve and dissolution curve, which are shown. **(C)** HeLa cells were infected with CVB3 (MOI = 10) and incubated for 6 h. Circ_0076631 levels were analyzed by RT-qPCR. *n* = 3, ***P < 0.01.

As previously noted, circ_0076631 regulated caspase-1 to mediate pyroptosis of diabetic cardiomyopathy (Yang et al., 2019). According to our previous study, pyroptosis is involved in the pathogenesis of both CVB3 and enterovirus A71 (EV-A71) (Wang Y. et al., 2018). Therefore we speculated that circ_0076631 may play role in regulating CVB3 replication. To clarify the source of hsa_circ_0076631 in human genome (hereafter simplified as circ_0076631), sequence analysis showed that circ_0076631 was originated from the transcript RNA of SLC29A1 between 155 nt (exon 3) and 1264 nt (exon 12) (Figure 1A). Ring formation validation indicated that circ_0076631 could form a ring, as indicated by the amplification curve and dissolution curve (Figure 1B). Upon real-time quantitative PCR (RT-qPCR) detection in the CVB3-infected cells for 6 h, the levels of circ_0076631 were increased, suggesting that circ_0076631 may be involved in CVB3 infection (Figure 1C).

### Circ_0076631 knockdown reduces CVB3 replication

**FIGURE 2:**
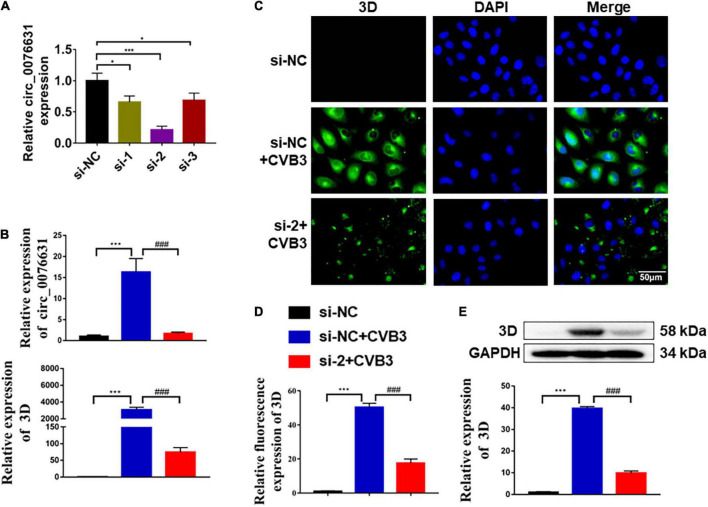
Circ_0076631 knockdown suppresses CVB3 replication. **(A)** HeLa cells were transfected with circ_0076631 siRNA (si-1, si-2, si-3) and cultured for 24 h. The abundance level of circ_0076631 was assayed by RT-qPCR. *n* = 3. *P < 0.05, ***P < 0.001. **(B–E)** HeLa cells were transfected separately with si-NC and si-circ-0076631 for 24 h. After infection with CVB3 (MOI = 10) for 6 h. **(B)** The relative abundance level of circ_0076631 and CVB3 3D-coding RNA detected by RT-qPCR. **(C,D)** Immunofluorescence staining was performed to detect the level of CVB3 3D protein. **(E)** The relative level of CVB3 3D protein was detected by western blotting. *n* = 3. **P < 0.01, ***P < 0.001 compared with the si-NC. #*P* < 0.05, ##*P* < 0.01, ###*P* < 0.001 compared with the si-NC + CVB3 group.

To evaluate the role of circ_0076631 in CVB3 infection, three siRNA against circ-0076631 (si-1, si-2, and si-3) were designed to knock down circ_0076631. RT-qPCR showed that three siRNAs worked out to knock circ_0076631 and si-2 demonstrated the highest efficiency (Figure 2A). In the cells transfected with si-2, after infection with CVB3 (MOI = 10) for 6 h, RT-qPCR (Figure 2B), immunofluorescence staining (Figures 2C,D), and western blotting (Figure 2E) showed that the levels of CVB3 3D RNA and protein were both decreased, suggesting that inhibition of circ_0076631 reduces CVB3 replication.

### Bioinformatics prediction of circ_0076631-binding miRNAs

**FIGURE 3:**
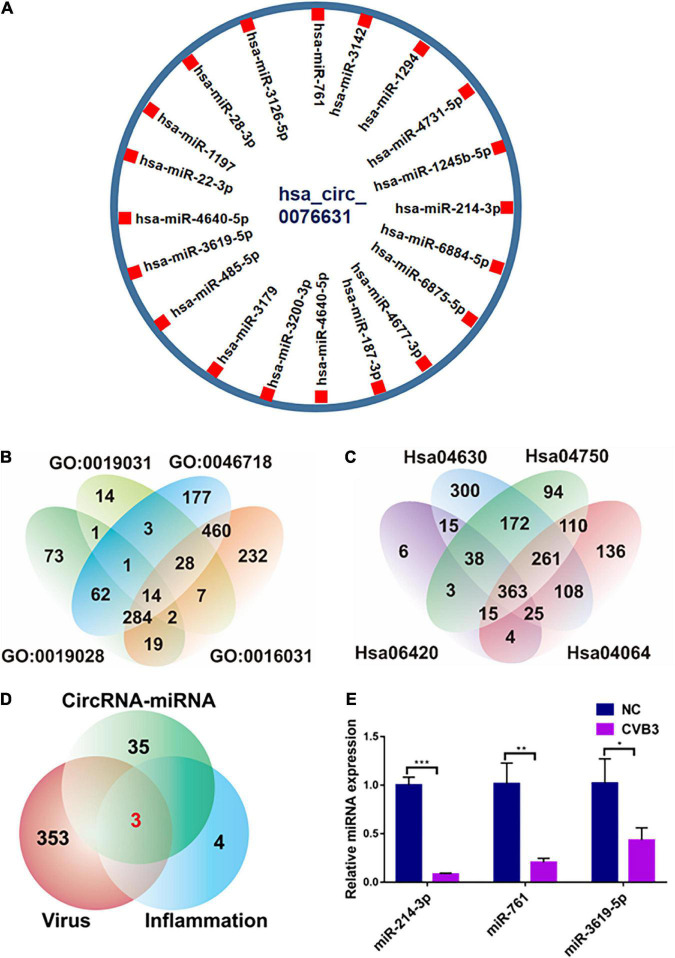
Prediction of miRNAs that may bind to targeting circ_0076631. **(A)** Screening the potential miRNAs that may bind to circ_0076631. **(B,C)** Screening of the miRNAs related to the inflammatory pathway and viral infection. **(D)** Comprehensive analysis of the three aforementioned screening results. **(E)** HeLa cells were infected with CVB3 (MOI = 10) for 6 h, and the abundance of miRNAs was measured by RT-qPCR. *n* = 3. *P < 0.05, **P < 0.01, ***P < 0.001.

To elucidate the mechanism of circ_0076631 regulating CVB3 replication, we searched for miRNAs that is capable of binding to circ_0076631 in the ENCORI database^1^ and found that 38 miRNAs could bind to circ_0076631 and the top 20 miRNAs were shown in Figure 3A. Then, we searched for miRNAs related to the inflammatory pathway and viral infection in the miRNAs database and mirPath v.3^2^ (Figures 3B,C). Three miRNAs were at the intersection of circ_0076631 binding, inflammation, and viral infection (Figure 3D). The detection of these miRNAs in HeLa cells infected with CVB3 showed that these miRNAs downregulated. Among them, miR-214-3p decreased most significantly (Figure 3E).

### Circ_0076631 can interact with miR-214-3p

**FIGURE 4:**
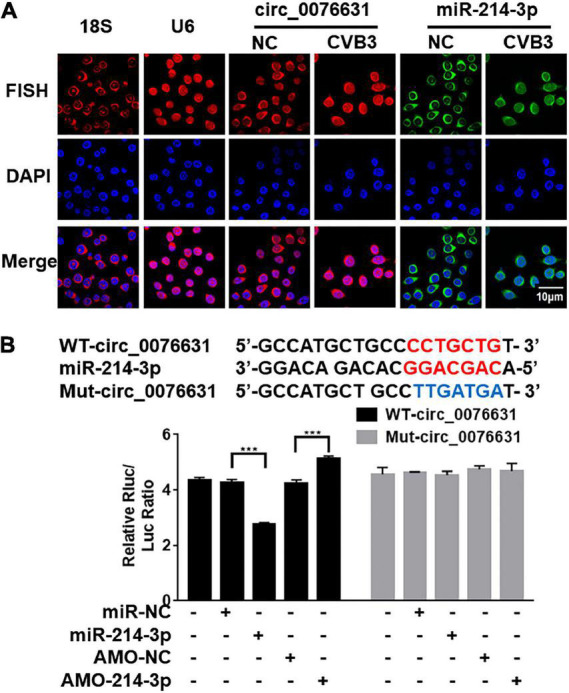
Verification of miR-214-3p binding to circ_0076631. **(A)** FISH assay was performed to observe the cellular localization of circ_0076631 in HeLa cells with CVB3 infection. Circ_0076631, 18S rRNA, U6 are showed in red. Nuclei are stained with DAPI (blue). **(B)** The miR-214-3p-binding sequence in wild-type circ_0076631 and the mutated circ_0076631. Plasmids expressing *Renilla* luciferase (Rluc) fused with wide-type circ_0076631 (containing miR-214-3p-targeting site, WT-circ_0076631) or with mutated circ_0076631 (without miR-214-3p-targeting site, Mut-circ_0076631) sequence were co-transfected with miR-214-3p mimic (miR-214-3p), miR-NC, AMO-miR-214-3p (AMO-214-3p), or AMO-miR-NC (AMO-NC) into HEK293T cells, respectively. Luciferase activity was detected with dual luciferase assay. The activity of Rluc was normalized with luciferase (Luc). *n* = 3. ***P < 0.001.

To detect the effect of CVB3 infection on the cellular localization of circ_0076631 and miR-214-3p, induced by CVB3, fluorescence *in situ* hybridization (FISH) assay demonstrated that circ_0076631 was in nucleus and cytoplasm, while miR-214-3p was in the cytoplasm of the non-infected cells. However, the distribution of circ_0076631 in the nucleus increased, while the distribution of miR-214-3p in cytoplasm of the CVB3-infected cells decreased (Figure 4A). To verify the interaction between circ_0076631 and miR-214-3p, the circ_0076631 cDNA was inserted into the 3′-UTR of *Renilla* luciferase (Rluc) gene of plasmid psiCHECK-2, denoted as WT-circ_0076631. Another plasmid with a mutated circ-007631 in which miR-214-3p-targeting site was removed was also prepared similarly and denoted as Mut-circ_0076631. HEK293T cells were transfected with the two plasmids respectively. Dual luciferase assay showed that the relative Rluc/Luc ratio was significantly decreased in the cells transfected with WT-circ_0076631 + miR-213-3p mimic, and increased in the cells transfected with WT-circ_0076631 + AMO-214-3p. No apparent change was observed in the cells with Mut-circ_0076631 treated with mimic or antisense miR-214-3p (AMO-214-3p), confirming that circ_0076631 can interact with miR-214-3p (Figure 4B).

### MiR-214-3p can target the 3D-coding sequence of CVB3

**FIGURE 5:**
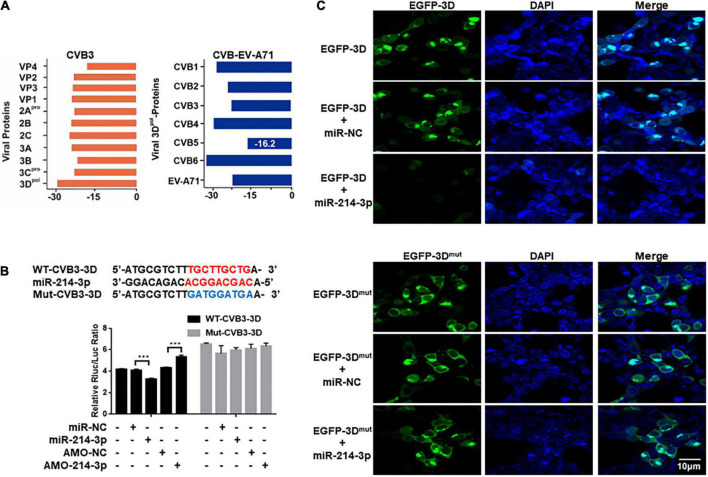
Prediction and verification of miR-214-3p tergetig CVB3 genomic RNA. **(A)** Identification of the potential miR-214-3p-binding sites in CVB3 genomic RNA by RNAhybrid. The binding ability of miR-214-3p with CVB1- CVB6 3D and EV-A71 3D sequence. **(B)** The miR-214-3p-binding sequence in the CVB3 3D-coding sequence and the mutated CVB3 3D sequence. Wide-type CVB3 3D sequence (containing miR-214-3p-binding sequence, WT-CVB3-3D) or mutated CVB3 3D sequence (without miR-214-3p-binding sequence, Mut-CVB3-3D) was cloned into a luciferase-expressing plasmid. The plasmid was co-transfected separately with miR-214-3p, miR-NC, AMO-214-3p, or AMO-NC into HEK293T cells, respectively. The luciferase activity was detected with dual luciferase assay. The acitivity of Rluc was normalized with Luc. *n* = 3. ***P < 0.001. **(C)** HEK293T cells were co-transfected with pEGFP-CVB3-3D (EGFP-3D) or pEGFP-CVB3-3D^mut^ (EGFP-3D^mut^) and miR-214-3p for 24 h. Immunofluorescence staining was performed to detect the expression level of the CVB3 3D protein.

Using miRNA target prediction tool RNAhybrid, miR-214-3p showed a strong binding capability to the 3D-coding sequence of CVB3 based on the minimum free energy hybridization (Figure 5A). Strong binding capability to the 3D-coding sequences of CVB1, CVB2, CVB3, CVB4, CVB6, and EV-A71 was also predicted (Figure 5A). According to the binding sequence, we constructed two dual luciferase reporter plasmids fused with a wildtype (WT-CVB3-3D) or a mutant (Mut-CVB3-3D) 3D-coding sequences of CVB3, respectively (Figure 5B). In the HEK293T cells transfected with WT-CVB3-3D, the relative Rluc/Luc ratio was significantly reduced when miR-214-3p mimics (miR-214-3p) was co-transfected, whereas the relative Rluc/Luc ratio remarkably increased when AMO-214-3p was given. The relative Rluc/Luc ratio remained no obvious change in the cells with Mut-CVB3-3D + miR-214-3p or Mut-CVB3-3D + AMO-214-3p (Figure 5B). To verify the target of miR-214-3p in the 3D-coding sequence of CVB3, HEK293T cells were co-transfected with miR-214-3p and a 3D-expressing plasmid (denoted as EGFP-3D) or a miR-214-3p binding-site mutated 3D-expressing plasmid (denoted as EGFP-3D^mut^). The EGFP-3D expression was almost invisible in the cells transfected with miR-214-3p, while EGFP-3D^mut^ expression was not affected by miR-214-3p transfection (Figure 5C).

### Circ_0076631 facilitates CVB3 infection through interacting with miR-214-3p

**FIGURE 6:**
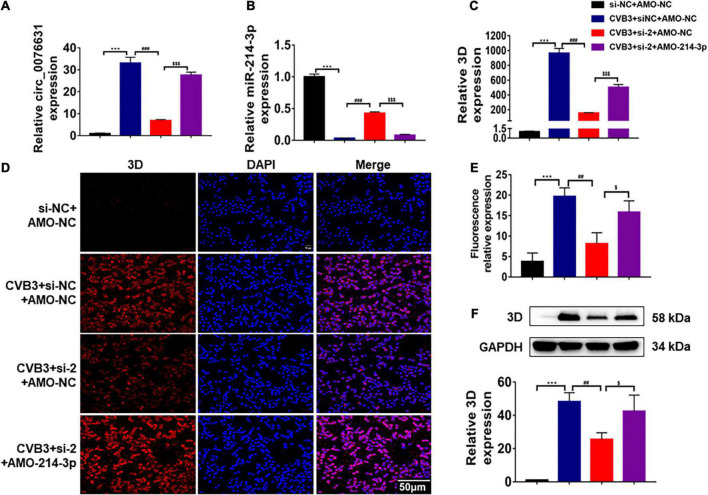
Circ_0076631 knockdown suppresses CVB3 replication through miR-214-3p targeting 3D-coding sequence. **(A)** HeLa cells were transfected separately with si-NC + AMO-NC, si-2 + AMO-NC, and si-2 + AMO-214-3p for 24 h, and infected with CVB3 (MOI = 10) for 6 h. **(A–C)** The relative abundance level of circ_0076631, miR-214-3p, and CVB3 3D was detected by RT-qPCR. **(D,E)** Immunofluorescence staining was performed to detect the level of the CVB3 3D protein. **(F)** The relative level of CVB3 3D protein was detected by western blotting. *n* = 3. ***P < 0.001 compared with the si-NC + AMO-NC group. ##*P* < 0.01, ###*P* < 0.001 compared with the CVB3 + si-NC + AMO-NC group. $*P* < 0.05, $$$*P* < 0.001 compared with the CVB3 + si-2 + AMO-NC group.

To evaluate the effect of circ_0076631 on CVB3 infection through miR-214-3p, HeLa cells were transfected with si-2 and infected with CVB3 (MOI = 10) for 6 h, RT-qPCR (Figures 6A–C), immunofluorescence staining (Figures 6D,E) and western blotting (Figure 6F) showed that the levels of CVB3 RNA and 3D protein were both decreased. The downregulation effect of si-2 could be mostly reversed by introducing AMO-214-3p. The data suggest that circ_0076631 facilitates CVB3 infection through interacting with miR-214-3p.

## Discussion

CircRNAs play critical roles in various disorders, including cancer, inflammation, fibrosis, and viral infection (Memczak et al., 2013; Zhang H. D. et al., 2018; Zhang Z. et al., 2018; Yang et al., 2019; Xie et al., 2021). Although there are studies reported that circRNAs participate in regulating viral infections (Chen et al., 2017; Li et al., 2017; Sekiba et al., 2018; Tagawa et al., 2018; Wang S. et al., 2018; Yu et al., 2019; Zhao et al., 2019; Lu et al., 2020; Zhang and Wang, 2020), it is unknown whether circRNA directly involves in regulating CVB infection. In this study, we found that circRNA circ_0076631 can promote CVB3 infection by interacting with miR-214-3p, possibly through miRNA sponging mechanism.

Over thousands of circRNAs have been identified in cells (Pamudurti et al., 2017; He et al., 2021). It is hard to select a single circRNA which may be functional in certain types of viral infections from such a large body of non-coding RNAs. In this study, we selected circ_0076631 based on our previous identification of circ_0076631 as a regulator of pyroptosis in diabetic cardiomyopathy (Yang et al., 2019).

Recent studies demonstrate that miRNAs are highly associated with CVB infection. MiR-10a*, miR-342-5p, miR-142-3p, miR-23b, and miR-296-5p can directly target enterovirus genomic RNA to promote or inhibit enterovirus replication (Kelly et al., 2010; Wang et al., 2012; Tong et al., 2013; Wen et al., 2013; Zheng et al., 2013). MiRNAs can also indirectly affect CVB infection, for example, miR-155 is recognized as a proinflammatory factor in CVB infection, knocking down miR-155 can improve the survival rate and cardiac function of the CVB-infected mice (Corsten et al., 2012). MiR-21 and miR-146b also participate in CVB infection, knocking down miR-21 and miR-146b can alleviate the CVB infection-related cardiac injury by decreasing the proportion of Th17 cells (Liu et al., 2013). We recently recognized miR-146a as an anti-inflammatory miRNA. MiR-146a expresses increasingly upon CVB infection and works as a host’s negative regulator on inflammation so as to restrain the inflammatory injury to an adequate scale (Fei et al., 2020). In this study, we found that miR-214-3p could directly interfere CVB3 replication by targeting the 3D-coding region. Though the target site is not in the 3′UTR, which miRNAs target most frequently, our loss-of-function test validated it as a viable target for miR-214-3p. Given that miR-10a*, miR-324-5p, miR-142-3p, and miR-296-5p, miR-23b are all target the coding region of enterovirus genome, it seems a common feature for enteroviruses that the coding region of enterovirus genome can be effectively targeted by miRNAs.

According to this study and a miRNA expression profiling we did previously (data not shown), miR-214-3p abundance undergoes a conversion from high abundant in the normal control cells to low abundant in the CVB-infected cells. Given the suppressing role of miR-214-3p to CVB, the conversion will favor CVB to replicate in the host cells. On the other hand, if the abundance of miR-214-3p can be artificially elevated, it may work as a therapeutic strategy against CVB infection.

**FIGURE 7:**
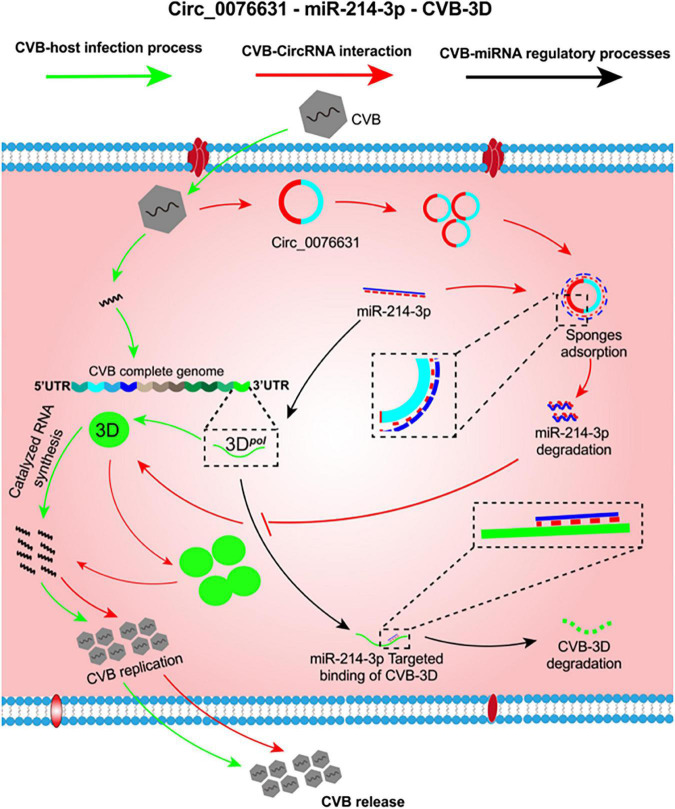
The putative mechanism of circ_0076631 regulating CVB3 infection by sponging miR-214-3p. At the early stage of CVB3 infection, miR-214-3p is abundant and can suppress viral biosynthesis by targeting the 3D-coding sequence (black lines). Later, CVB3 infection can somehow trigger circ_0076631 generation. Circ-0076631 facilitates CVB3 replication through sponging miR-214-3p and relieving its suppression on CVB3 biosynthesis (red lines).

The decrease of miR-214-3p abundance in the CVB-infected cells can be an outcome of failures such as downregulated transcription of primary miRNA, impedimental maturation process, and removal by degradation or sponge absorption. CircRNAs can sponge miRNAs to reduce their availability and thus reduce their binding to target mRNAs. The possibility of sponge absorption led us to probe the potential role of circRNAs. Through a bioinformatics searching for circRNAs that may interact with miR-214-3p, circ_0076631 was selected because it owns an 8-nt complemental sequence to miR-214-3p, and it became abundant in the cells with CVB3 infection. Our further evaluation supported that miR-214-3p can bind to circ_0076631. Knockdown of circ_0076631 could inhibit CVB3 infection. The inhibition could be relieved by AMO-214-3p. Taken together, we hypothesize that CVB3 infection upregulates the expression of circ_0076631, which then sponges miR-214-3p to relieve its suppression on CVB3 biosynthesis by targeting the 3D-coding sequence. To date, little is known about the association between circRNAs and enterovirus infection. CircSIAE is an exception that it can act as a sponge for miR-331-3p and inhibit CVB3 infection indirectly by up-regulating host’s TAOK2 expression (Yang et al., 2022). Our finding provides another example of circRNA interfering enterovirus infection in a much more direct way. Furthermore, the target site of miR-214-3p is available in CVB1, CVB2, CVB3, CVB4, CVB6, and EV-A71, the regulation of circ_0076631/miR-214-3p is likely applicable to most serotypes of CVB and other enteroviruses. On the basis of the aforementioned data, a putative mechanism is summarized in Figure 7.

There is limitation in this study. The conclusion about the interaction between circ_0076631/miR-214-3p and CVB infection is based solely on *in vitro* data. Considering that CVB is the major pathogen of human viral myocarditis and DCM (Tian et al., 2018), it is a shortage of the study that the role of circ_0076631 in the myocardial pathogenesis of CVB has not been evaluated. *In vivo* investigation is needed to address the question. Unfortunately, mouse model with circ_0076631 knockdown or knockout is not available presently. In addition, only one serotype (CVB3) has been evaluated experimentally, the interaction between circ_0076631 and other serotypes of CVB is yet to be observed.

In summary, circ_0076631 may play a supportive role in CVB3 infection. Circ_0076631 facilitates CVB3 infection by sponging miR-214-3p. Knocking down circ_0076631 or giving miR-214-3p may be a potential approach to treat CVB3 infection.

## Materials and methods

### Cell culture

Human cell lines including HeLa, HEK-293T were cultured in Dulbecco’s modified Eagle medium (DMEM) (HyClone, Logan, UT, United States) containing 10% fetal bovine serum (FBS; Gibco, Scoresby VIC, Australia), penicillin (100 U/mL) and streptomycin (0.1 mg/mL). The cells were incubated at 37°C in 5% CO_2_ and passaged every 48 h.

### Viruses and viral infection

CVB3 Woodruff strain was cultured in HeLa cells. The virus titer was measured by the plaque-forming unit (pfu) as described previously (Tong et al., 2013). The titer of CVB3 was 1 × 10^8^ pfu/mL and a multiplicity of infection (MOI) of 10 was used to infect cells.

### Transfection

Nucleotides, including short interfering RNA (siRNA) of circ_0076631, miR-214-3p mimic (miR-214-3p), AMO-214-3p (mAmCmUmGmCmCmUmGmUmCmUmGmUmGmCmCm UmGmCmUmGmU), miR-mock, and AMO-mock were synthesized by RiboBio (Guangzhou, China). HEK293T cells were transiently transfected with the aforementioned nucleotides using X-Treme GENE transfection reagent (Roche, Mannheim, Germany) according to the manufacturer’s instructions. In brief, cells were cultured in 12-well plates until they reached 60–75% confluence. The culture medium was then replaced with serum-free medium and transfected with 50 μL of transfection mixture. After incubation for 4 h, the transfection mixture was discarded, and fresh medium with 10% FBS and antibiotics were added to the cells and maintained at 37°C with 5% CO_2_.

### Construction of mutant 3D-expressing plasmid

A plasmid expressing CVB3 3D with a mutated miR-214-3p-binding site was constructed based on pEGFP-3D, a plasmid expressing CVB3 3D fused with enhanced green fluorescence protein (EGFP) engineered previously in our lab. Briefly, mutant pEGFP-3D was prepared with the mutation of nt6977-nt6985 in CVB3 genome by overlapping PCR. The restriction sites of *Hin*d III and *Xba* I were added to both ends of pEGFP-3D with specific primers. After digestion with (TaKaRa, Otsu, Shiga, Japan), the mutant 3D fused with EGFP was inserted to the multiple cloning site of pEGFP-C1 and designated as pEGFP-3D^mut^.

### Reverse transcription and real-time quantitative PCR

Total RNA samples were extracted with TRIzol (Invitrogen, Carlsbad, CA, United States) according to the manufacturer’s instructions. After detecting the purity and concentration of the RNA with a NanoDrop2000 spectrophotometer (Thermo Fisher, Waltham, MA, United States), 1 μg of total RNA was used as a template with PrimeScript RT Enzyme Mix I (TaKaRa, Otsu, Shiga, Japan) in a reverse transcription system. RT-qPCR was performed on a LightCycler 96 (Roche, Basel, Switzerland) using SYBR Premix Ex Taq II (TaKaRa, Otsu, Shiga, Japan) mixed with 1 μL of the synthesized cDNA and sense/antisense primers to a final volume of 25 μL. The primer sequences used in this study are listed in Table 1. The relative gene expression of the circRNAs, miRNAs and mRNAs level was calculated by the 2^–ΔΔCt^ method (Livak and Schmittgen, 2001). The expression of circ_0076631, miR-214-3p was normalized to that of U6, and the expression of the other sequences was normalized to that of glyceraldehyde-3-phosphate dehydrogenase (GAPDH).

**TABLE 1:** PCR primer sequences.

Primer		Sequence
hsa_circ_0076631	Forward	5′-CGGAGCCTCACAGCTGTATTC-3′
	Reverse	5′-CCCAGACCCAGCATGAAGAAG-3′
miR-214-3p	Forward	5′-TATACATCAAACAGCAGGCACA-3′
	Reverse	5′-CATTCGATCTTCTCCACAGTCTC-3′
CVB 3D	Forward	5′-TCATGACACCAGCAGACAAA-3′
	Reverse	5′-TCCTTGGTCCATCTGATTGA-3′
GAPDH	Forward	5′-ATCACTGCCACCCAGAAGAC-3′
	Reverse	5′-TTTCTAGACGGCAGGTCAGG-3′
U6	Forward	5′-CTCGCTTCGGCAGCACATATACT-3′
	Reverse	5′-ACGCTTCACGAATTTGCGTGTC-3′

### Western blotting

Total protein was extracted with RIPA lysis buffer (Thermo Scientific, Rockford, United States) mixed with phenylmethane sulfonyl fluoride (PMSF). The protein samples were separated by 10% sodium dodecyl sulfate-polyacrylamide gel electrophoresis (SDS-PAGE), and the proteins were transferred onto the PVDF membrane (Millipore, Billerice, MA, United States). Then, the membrane was blocked with 5% non-fat milk dissolved in TBST for 1 h at room temperature and subsequently incubated with the corresponding primary antibodies overnight at 4°C, followed by HRP-conjugated secondary antibodies for 1 h at room temperature. The membranes were imaged with FluorChem M CCD camera (ProteinSimple, Santa Clara, CA). GAPDH was used as the internal control. The primary antibody against GAPDH was purchased from ZSGB-BIO (Beijing, China). A polyclonal antibody against 3D protein of CVB3 was generated in our laboratory.

### Immunofluorescence staining

Cells plated on coverslips were fixed with 4% paraformaldehyde in PBS for 30 min at room temperature. Then, the cells were permeabilized with 0.1% Triton X-100 for 1 h followed by blocking with 500 μL of goat serum for 2 h at 37°C. Then cells were incubated with primary antibodies against 3D (1:200) at 4°C overnight. After three washes, the cells were incubated with FITC-conjugated secondary antibody (1:1,000) for 1 h at 37°C. Then, the nuclei were stained with DAPI (Beyotime, Shanghai, China) for 20 min. Images were collected with a fluorescence microscope (AxioVert 200, Zeiss).

### MiRNA target prediction and verification

To predict the targets of miRNAs, we used RNAhybrid 2.2^3^ and selected the potential binding sites according to the minimum free energy (mfe) of complementary base sequences. A luciferase reporter was constructed with the putative target sequences of miR-214-3p to verify the target effects (RiboBio, Guangzhou, China).

### Luciferase assays

The corresponding linear sequence of circ_0076631 was obtained with circBase software, and dual-luciferase plasmids including circ_0076631 with miR-214-3p-binding sites (WT-circ_0076631) or with a mutated sequence to remove miR-214-3p-binding site (Mut-circ_0076631) were constructed by RiboBio. Similarly, dual-luciferase plasmids including CVB3 3D sequence with with miR-214-3p-binding sites (WT-CVB3-3D) or a mutated sequence to remove miR-214-3p-binding site (Mut-CVB3-3D) were constructed. The wild-type or mutated plasmids with miR-214-3p or AMO-214-3p were transfected into HEK293T cells by Lipofectamine 2000, respectively. Luciferase activity was tested using a Dual-Luciferase Reporter assay (Promega).

### Fluorescence *in situ* hybridization

To observe the location of circ_0076631, HeLa cells were cultured in 12-well plates for 24 h until they reached 60–70% confluency. Cells with or without CVB3 infection were fixed in 4% paraformaldehyde and permeabilized in 0.5% Triton X-100. The cell samples were treated with re-hybridization solution and incubation. A hybridization buffer containing a Cy3-labeled FISH probe for detecting circ_0076631 and the internal controls 18 s and U6 were used to replace re-hybridization solution and added into cell samples which were incubated at 37°C overnight in the dark. After washing with sodium citrate (SCC) and PBS, DAPI was added to the samples for nuclear staining. Then, the samples were treated with a mounting medium and observed by fluorescence microscope (AxioVert 200, Zeiss).

### Statistical analysis

All data in this study are presented as the means ± standard deviation (SD) and determined via GraphPad Prism 7 software to perform statistical analysis. Student’s *t-*test or one-way ANOVA was used to evaluate the significance of difference. All experiments were repeated at least three times.

## Data availability statement

The original contributions presented in the study are included in the article/supplementary material, further inquiries can be directed to the corresponding authors.

## Ethics statement

The animal study was reviewed and approved by the Ethics Committee of Harbin Medical University.

## Author contributions

YQ, LL, and SY conceived and designed the project. YQ, JH, FD, and XY performed the experiments. SY and LR assisted in the collection of data. ZD, CZ, LR, and YF analyzed and interpreted the data. LL wrote the manuscript. YW, ZZ, and WZ reviewed and critically revised the initial draft. All authors contributed to the article and approved the submitted version.

## Footnotes

1
https://starbase.sysu.edu.cn/agoClipRNA.php?source=lncRNA

2
https://dianalab.e-ce.uth.gr/html/mirpathv3/index.php?r=mirpath

3
https://bibiserv.techfak.uni-bielefeld.de/rnahybrid
